# Steady-like topology of the dynamical hydrogen bond network in supercooled water

**DOI:** 10.1093/pnasnexus/pgac090

**Published:** 2022-06-17

**Authors:** Fausto Martelli

**Affiliations:** IBM Research Europe, Keckwick Lane, Daresbury, WA4 4AD, UK

**Keywords:** network topology, supercooled water, glassy water, large-scale density fluctuations

## Abstract

We investigate the link between topology of the hydrogen bond network (HBN) and large-scale density fluctuations in water from ambient conditions to the glassy state. We observe a transition from a temperature-dependent topology at high temperatures, to a steady-like topology below the Widom temperature *T_W_* ∼ 220 K signaling the fragile-to-strong crossover and the maximum in structural fluctuations. As a consequence of the steady topology, the network suppresses large-scale density fluctuations much more efficiently than at higher temperatures. Below *T_W_*, the contribution of coordination defects of the kind *A*_2_*D*_1_ (two acceptors and one donor) to the kinetics of the HBN becomes progressively more pronounced, suggesting that *A*_2_*D*_1_ configurations may represent the main source of dynamical heterogeneities. Below the vitrification temperature, the freezing of rotational and translational degrees of freedom allow for an enhanced suppression of large-scale density fluctuations and the sample reaches the edges of nearly hyperuniformity. The formed network still hosts coordination defects, hence implying that nearly hyperuniformity goes beyond the classical continuous random network paradigm of tetrahedral networks and can emerge in scenarios much more complex than previously assumed. Our results unveil a hitherto undisclosed link between network topology and properties of water essential for better understanding water’s rich and complex nature. Beyond implications for water, our findings pave the way to a better understanding of the physics of supercooled liquids and disordered hyperuniform networks at large.

Significance Statement We expose a hitherto undisclosed link between topology of the hydrogen bond network (HBN) and properties of water, and we identify a coordination defect as the source of dynamical heterogeneities. The HBN in supercooled liquid water preserves a temperature-independent topology that efficiently suppresses large-scale density fluctuations. The steady topology of the network implies the existence of collective bonds transformations with long-range correlations. Hyperuniformity, a state of matter that suppresses large-scale density fluctuations, can be accommodated in networks hosting as much as 20% coordination defects, hence going beyond the paradigm of continuous random networks. Our results enrich our understanding of the complex properties of water and supercooled liquids, and pave the way to a deeper understanding of nearly hyperuniform networks.

## Introduction

The simple molecular structure of water hides a remarkably wide list of anomalous behaviors that stretch over the most complex phase diagram of any pure substance (1), and whose origin lies in a critical point located at low temperatures and low pressures (2–6), in a region of the phase diagram known as “no-man’s land.” At these thermodynamic conditions, below the line of homogeneous ice nucleation, supercooled water undergoes very rapid crystallization (7, 8), on time scales too short for experiments to accurately probe the liquid phase, from which the epithet “no-man’s land.” The absence of an experimental proof of the existence of a liquid–liquid critical point (LLCP) led to contrasting interpretations of numerical results sparking heated debates that lasted for several years (2,9–13); the major criticism moved against the existence of a LLCP was the possibility that observations of the transition between two liquid forms were due to undetected crystalline structures (11, 12). Later simulations discarded such possibility (14) and showed that arguments against the LLCP scenario were based on imprecise sampling of the rotational degrees of freedom in the liquid phase (15). More recently, further proofs of the validity of the LLCP scenario have been reported from computational investigations of realistic models of water (4), and experimental efforts have pushed the boundaries of the no-man’s land providing important evidences supporting the existence of the critical point (5, 6). From the LLCP, a bundle of lines emanates in the phase diagram towards the more familiar regions of ambient conditions; such lines, commonly known as Widom lines, are the loci of maxima of thermodynamic response functions which tend to diverge upon approaching the LLCP. The maxima in thermodynamic response functions are strictly linked to the spatial arrangement and clustering of water molecules into ordered and disordered local environments and their dynamical properties (16, 17).

At ambient pressure, upon cooling, liquid water undergoes a fragile-to-strong transition occurring at *T_W_* ∼ 220 K (18). This dynamical transition is connected to the percentage of hydrogen-bonds that gradually build up upon cooling (19,20) and signals the breakdown of the Stokes–Einstein relationship crossing over from a non-Arrhenius (fragile) to an Arrhenius (strong) behavior (21). The breakdown of the Stokes–Einstein relationship seems to be related to the development of dynamical and spatial heterogeneities that cause a decoupling of rotational and translational degrees of freedom, with the development of clusters of molecules rotating faster than the average (22–24).

At deeply supercooled conditions, water exhibits polyamorphism, i.e. it can exist in more than one glassy state. Indeed, the most common forms of glassy solid water are the low-density and high-density amorphous ices (25–34), which are connected by a first-order phase transition (25, 26, 35–38) and which are structurally linked to the liquid phases at equilibrium conditions (39). Moreover, the complex behavior of glassy solid water is reflected in the fact that amorphous ices seem to encompass a larger set of subfamilies characterized by different structural properties (40–44). Recently, it has been shown that amorphous ices are able to suppress, up to some extent, large-scale density fluctuations (37, 38) and are, therefore, nearly hyperuniform. Central to the concept of hyperuniformity (45) is the structure factor *S*(**k**). In the thermodynamic limit, $S(\mathbf {k})=1+\rho \tilde{h}(\mathbf {k})$, where $\tilde{h}(\mathbf {k})$ is the Fourier transform of the total correlation function (pair correlation function minus one) and $\bf k$ is the wavevector. The vanishing of normalized long-range density fluctuations in hyperuniform systems implies that *S*(*k*) → 0 for *k* → 0, where $k=|\mathbf {k}|$ is the wavenumber and *S*(*k*) is the structure factor $S(\mathbf {k})$ averaged over all directions at wavevector $\bf k$. A useful practical measure of the degree of hyperuniformity in a system is provided by the hyperuniformity index $H\equiv \frac{S(0)}{S(k_{peak})}$, where *k_peak_* is the wavenumber *k* at which *S*(*k*) has its maximal peak value. It is worth to remark that, in the thermodynamic limit, *S*(*k*) is related to the isothermal compressibility *k_T_* via the relation *S*(0) = *k_T_*ρ*k_B_T*, being ρ the density and *k_B_* the Boltzmann constant. Crystals, for which *S*(0) = 0, and hence *H* = 0, are trivially hyperuniform: being uncompressible, they are devoid of large-scale density fluctuations. On the other hand, disordered systems in which *H* ∼ 10^−3^ or smaller are deemed to be nearly hyperuniform (46) and are characterized by the appearance of some degree of long-range correlations. Hyperuniformity in disordered systems is linked to an increased packing efficiency with respect to random distributions, and many natural systems, from the structure of the early universe (47) to prime numbers (48) to biological systems (49,50), find such configurations to be efficient arrangements (51). The reference state that is used in the definition of *H*, i.e. the the normalization of *S*(0) with respect to *S*(*k_peak_*), derives from how the scattering intensity evolves as density increases from a low-density phase to a perfectly hyperuniform state. At very low densities, the scattering pattern is very uniform because the particles are spatially uncorrelated, but as the density increases, there is increasingly less scattering around the origin and increasingly a dominant higher-intensity concentric ring emerges around the origin located in the vicinity of wavenumber *k_peak_*. Thus, the value *S*(*k_peak_*) is an important reference state to be compared to *S*(0).

The network topology in disordered materials is strongly related to $S(\mathbf {k})$ and is an important structural descriptor for understanding the nature of disorder that is usually hidden in pairwise correlations. Correlations between dynamics, anomalous behaviors, and hydrogen bond network (HBN) have been reported in the case of water (16,52), but a direct link between the topology of the network (the geometric configurations of the network) and the degree of suppression of large-scale density fluctuations is still a missing piece of information, crucial for a deeper understanding of the properties of network forming materials at large.

In this article, we quantify the large-scale density fluctuations of liquid water and of glassy water modeled via classical molecular dynamics simulations and we link it to the topology of the HBN measured via the ring statistics, a theoretical tool that has been instrumental in understanding the properties of water at different thermodynamic conditions (2, 16, 36, 52–61). We show that, in correspondence with the fragile-to-strong transition, the topology of the HBN becomes steady and the corresponding fluctuations strongly damped, implying that the kinetics of the HBN at supercooled conditions is governed by collective rearrangements with long-range correlations. Moreover, we show that the decoupling between translational and rotational degrees of freedom occurring in correspondence with the fragile-to-strong transition is linked to the activity of coordination defects of the kind *A*_2_*D*_1_, in which a water molecule accepts two HBs and donates one.

Our results shed light on the complex nature of water and on the delicate balance between structural and dynamical properties developing at supercooled conditions, with possible applications to other network forming materials at large.

## Methodology

We have performed out-of-equilibrium classical MD simulations of a sample of *N* = 50,000 water molecules interacting with the TIP4P/2005 potential (62) in the NPT ensemble. We have employed Nosé–Hoover thermostat (63, 64) with 0.2 ps relaxation time to maintain constant temperature, and Parrinello–Rahman barostat (65) with 2 ps relaxation time to maintain constant pressure. We have performed simulations with the GROMACS 18.0.1 package (66). We have truncated short-range interactions at 9.5 Å, and we have computed long-range electrostatic terms using particle mesh Ewald with a grid spacing of 1.2 Å. We have equilibrated liquid water at *T* = 300 K and simulated the cooling and vitrification of the sample implementing a quenching rate *q_c_* = 1 K/ns until the final temperature of *T* = 100 K is reached. All results are the average of five independent simulations. Following Giovambattista et al. (35), we simulate the vitrification of our samples cooling liquid water from ambient temperature to *T* = 100 K at a quenching rate *q_c_* = 1 K/ns. While different quenching rates produce glassy water on different regions of the potential energy landscapes (35,38, 67), the overall conclusions of this work are transferable to such other scenarios.

In order to probe the topology of the HBN we have adopted the ring statistics. Starting from a water molecule, we construct rings recursively traversing the HBN until the starting point is reached again or the path exceeds the maximal ring size considered (12 water molecules in our case). We do not distinguish between the donor/acceptor character of the starting water molecules (53).

## Results

**Fig. 1:**
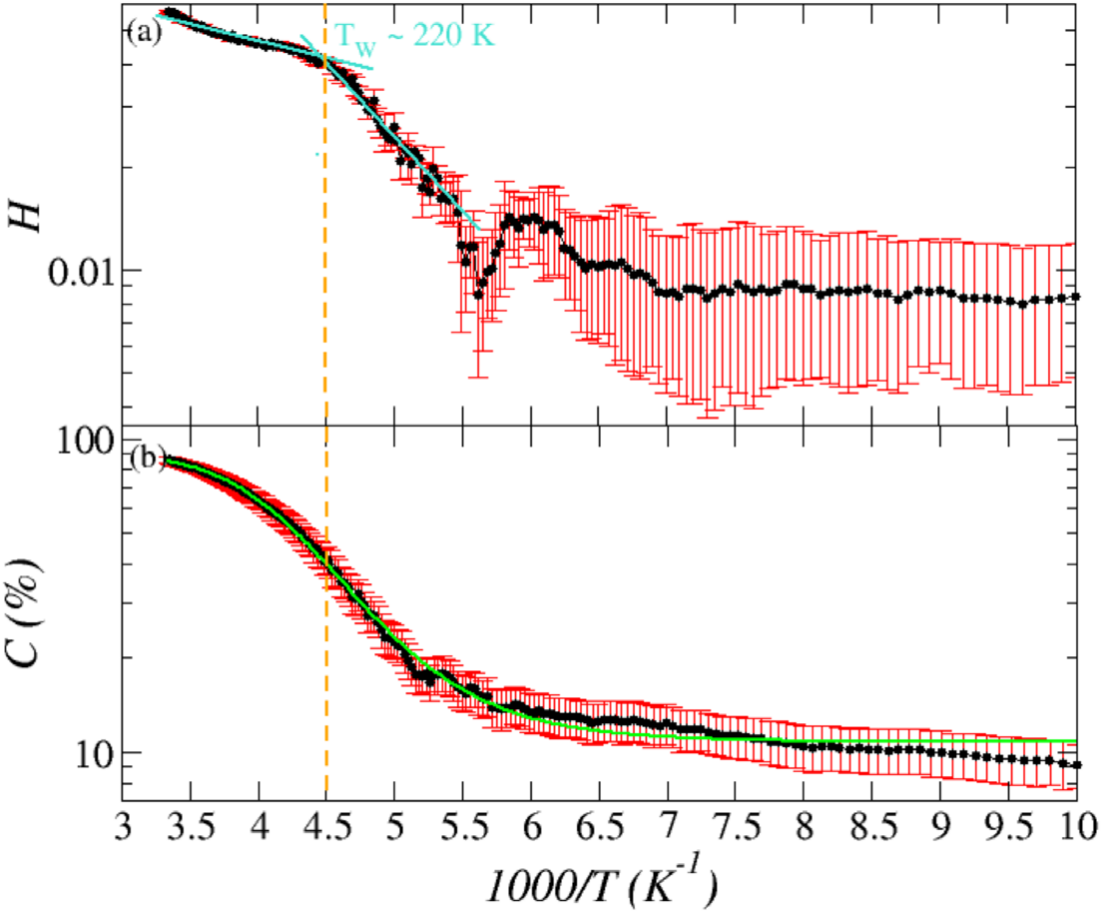
Panel (a): *H* as a function of the 1/*T* upon cooling the liquid sample from *T* = 300 K to *T* = 100 K. The cyan straight lines emphasize the presence of the crossover occurring at *T_W_* and are obtained from fitting the data points below and above *T_W_*. Panel (b): profile of *C* as a function of 1/*T* and computed as follows. At a given time step, we compute the list of water molecules that are hydrogen bonded to a target water and we compute the number of entries in the list of neighbors that differ from the previous time step. Therefore, *C* represents the percentage of water molecules changing one or more hydrogen bonded neighbor in the time window between *t* and *t* + *dt*, with *dt* = 1 ps. The green line represents the fit with a four-points logistic function.

In Fig. 1 we report the profiles of the hyperuniformity index *H* and of the coefficient *C* measuring the percentage of water molecules that change at least one hydrogen bonded neighbors in the time window of 1 ps. At temperatures close to ambient, the value of *H* reported in Fig. 1(a), only slightly decreases upon decreasing the temperature, as expected for a system in the fluid phase pervaded by large-scale density fluctuations. A clear crossover occurs roughly in correspondence with the Widom temperature (*T_W_* ∼ 220 K, or 1000/*T* = 4.5/K), which signals the maximum *k_T_* and in structural fluctuations, (39, 68–70) and the onset of the fragile-to-strong crossover (18, 19). Below *T_W_*, we observe a remarkable increase in slope of the values of *H* indicating that the supercooled *liquid*—although still hosting large-scale density fluctuations—is endowed with an enhanced capability to dampen large-scale density fluctuations. In the following discussion, we shall see that this remarkable enhanced efficiency is linked to the topology of the HBN and its fluctuations.

For this model of water, at the simulated quenching rate which is two order magnitude faster than the estimated minimum experimental rate (71), vitrification occurs at *T_v_* ∼ 200 K (35,37, 38, 72), ∼60 K above the experimental data (71, 73). The value of *H* continuously decreases while decreasing the temperature from *T_W_* down to *T* ∼ 180 K, corresponding to 1,000/*T* ∼ 5/K. Upon further cooling we observe an initial small increase in the value of *H*, which eventually stabilizes at *H* ∼ 8 × 10^−3^. We posit that the increase in the value of *H* in the temperature window 1,000/*T* ∈ [∼5.5 − 6.0] K^−1^ is indicative of a rearrangement occurring at the level of the HBN, which induces large-scale density fluctuations. Considering the error on the measure, we can qualitatively state that glassy water is nearly hyperuniform, in agreement with previous investigations (37,38).

In Fig. 1(b), we report the percentage of water molecules that change one or more hydrogen bonded neighbor on a time window of 1 ps. The value of $C\sim 90\%$ at ambient temperature indicates that ${\sim}90\%$ of water molecules change one or more hydrogen bonded neighbor every ps. As translational and rotational degrees of freedom become progressively less accessible upon cooling, *C* decreases accordingly. Such decrease follows the typical *S*-shaped profile of logistic functions describing populations dynamics, like surface growth, bacteria population, deposition of materials, clusters growth, and so on (74). The logistic function fitting our computational data is reported in Fig. 1(b) as a green continuous curve. Remarkably, the flex of the logistic function, defining where the rate of change switches sign, is located in correspondence with *T_W_*, hence proving that the fragile-to-strong transition is intimately connected to the dynamics of the HBs.

Dynamical arrest occurs at *T_v_* = (1,000/*T*) ∼ 5. Although rotational and translational degrees of freedom are frozen below *T_v_*, thermal fluctuations allow water molecules to change their connectivity through localized vibration, implying that the HBN is a dynamic system that keeps rearranging. Nearly hyperuniformity emerges and the value of *C* decreases very slowly, reaching ${\sim}10\%$ at 1,000/*T* = 10/K. As we will see in the forthcoming discussion, the requirement for nearly hyperuniformity to be accommodated in dynamic networks is that the overall network topology is preserved.

**Fig. 2:**
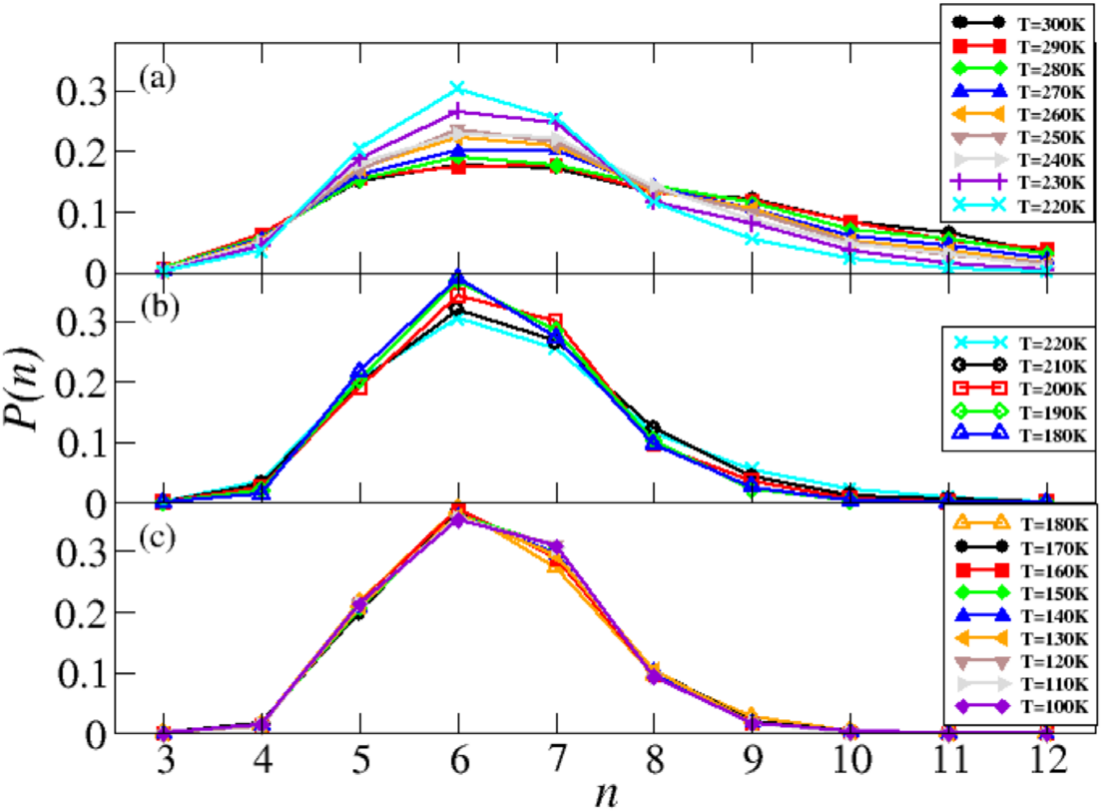
Probability *P*(*n*) of having a ring of length *n* as a function of the temperature. Panel (a) reports *P*(*n*) in the temperature window *T* ∈ [300 − 220] K corresponding to the slow decrease in the index *H*. Panel (b) shows *P*(*n*) in the temperature window *T* ∈ [220 − 180] K corresponding to the enhanced reduction in the index *H*. Panel (c) reports *P*(*n*) in the temperature window *T* ∈ [180 − 100] K in correspondence of the emergence of nearly hyperuniformity.

In Fig. 2 we report *P*(*n*), the normalized probability of having a ring of a given length *n*. Panel (a) reports the distribution *P*(*n*) computed in the temperature window *T* ∈ [300 − 220] K, corresponding to the temperature window in which the liquid sample is pervaded by large-scale density fluctuations and *H* decreases very slowly decreasing the temperature (Fig. 1a). At *T* = 300 K, the distribution *P*(*n*) is very broad, with an almost equal contribution of heptagonal (*n* = 7) and hexagonal (*n* = 6) rings, being *n* = 6 the configuration of the ground state which, at ambient pressure, are cubic and hexagonal ices. Both *n* = 6 and *n* = 7 account for ${\sim}17\%$ of the overall topology. The network is also rich in pentagonal rings (*n* = 5), which account for ${\sim}15\%$ of *P*(*n*), while *n* = 8 and *n* = 9 for ${\sim}13\%$ and ${\sim}12\%$, respectively. The network accommodates also a non-negligible percentage of longer rings, namely *n* = 10 and *n* = 11, and a smaller percentage of *n* = 12 and squared rings (*n* = 4). Upon cooling down to *T* = 220 *K* (cyan symbols, corresponding to the Widom temperature) we observe a gradual—but consistent—depletion of longer (*n* ≥ 9) rings from the network and a corresponding increase in shorter rings, especially in *n* = 6. This reshaping of the HBN toward topologies richer in *n* ∈ [5 − 7] and poorer in longer ones is a consequence of the reduction of translational and rotational degrees of freedom, which cause a contraction of the available configurational space and, overall, of the configurational entropy.

Panel (b) reports one of the main results of this work, namely the distribution *P*(*n*) in the temperature window *T* ∈ [220 − 180] K, i.e. in the temperature range in which the supercooled liquid shows a enhancement in suppressing large-scale density fluctuations as marked by the rapid decrease in *H* (Fig. 1a). Remarkably, upon cooling below *T_W_*, the overall topology of the HBN stabilizes towards a steady topology—in terms of preserved percentage of *n*-member rings—and does not show any significant modification, but only a mild enhancement in the contribution of *n* = 6 occurring mostly at *T* = 180 K. In this temperature range, the networks are dominated by *n* ∈ [5 − 8], with only a minor contribution of *n* = 4 and *n* = 9, and are mostly deprived of longer (*n* > 9) rings. We posit that the absence of longer rings is another key factor in the enhanced suppression of large-scale density fluctuations as longer rings account for higher densities. (16,53, 60).

**Fig. 3:**
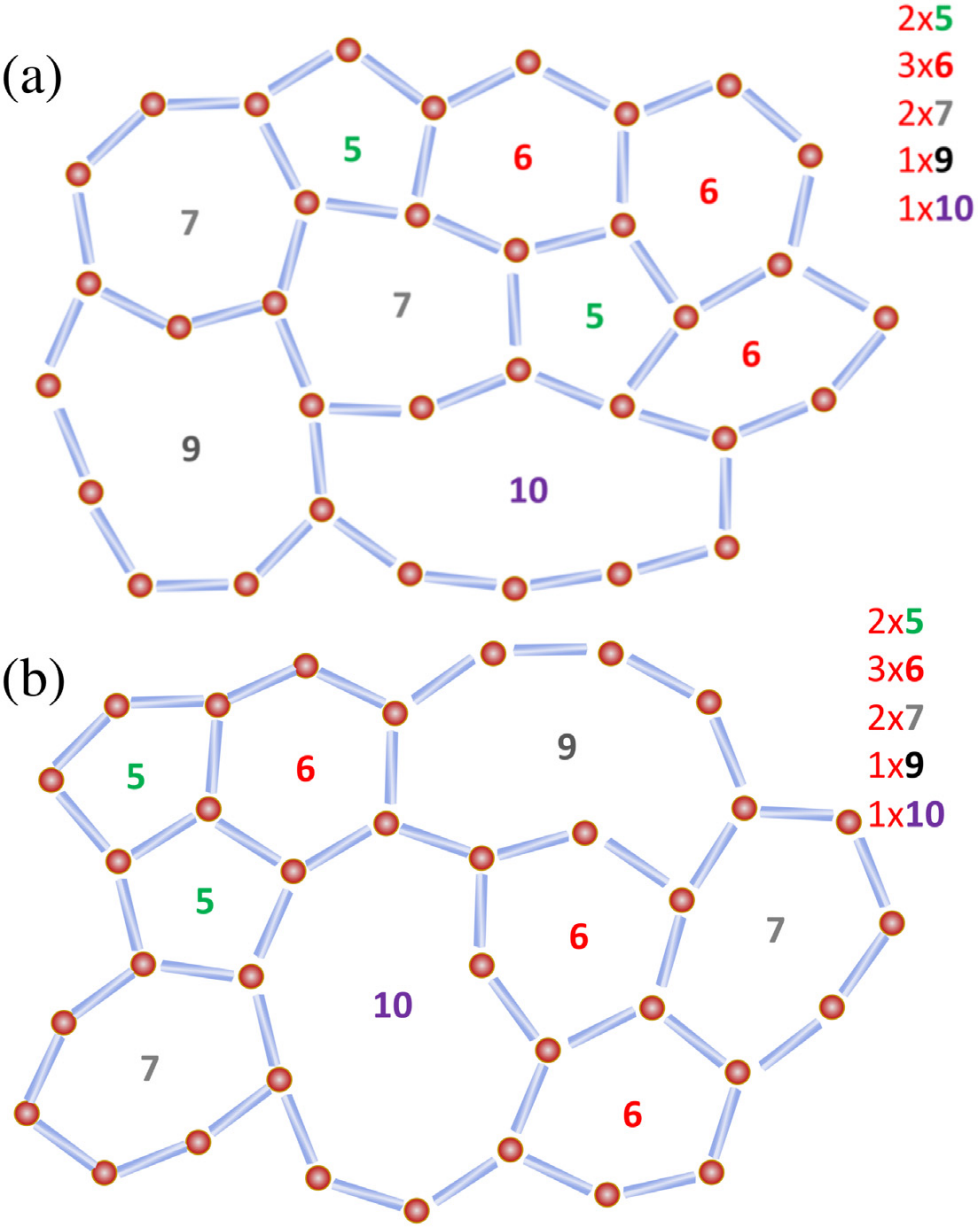
Pictorial representations of two snapshots at two different time steps. Water molecules are depicted as red spheres, and bonds with sticks. The connectivity moving from panel (a) to panel (b), corresponding to two consecutive time steps, has changed, but the overall topology is preserved. This snapshot reports the specific case in which the total number of rings is conserved.

Considering that the sample is *liquid* and ${\sim}50\%$ of water molecules changing one or more HBs every ps at *T_W_* and ${\sim}20\%$ at *T* = 180 *K* (Fig. 1b), the steady topology of the HBN is a remarkably surprising property that boosts the sample’s efficiency in suppressing large-scale density fluctuations. Such steady topology implies that the underlying kinetics governing networks transformations is strongly correlated and of long-range in nature. A pictorial representation of this effect is reported in Fig. 3, showing two snapshots with different connectivity but the same topology and, therefore, the same distribution *P*(*n*).

The striking evidence of a steady-like topology of the HBN in supercooled *liquid* water allows us to conclude that the dynamics of the HBN undergoes a transition from a uncorrelated kinetics at *T* > *T_W_* where Stokes–Einstein relationship holds, to a correlated one, at *T* < *T_W_* where Stokes–Einstein relations breaks down. Rationalizing the correlated nature of bond breaking / making at *T* < *T_W_* is a beguiling nontrivial task on which we will try to shed light in the forthcoming discussion.

Panel (c) reports the distribution *P*(*n*) in the temperature range *T* ∈ [180 − 100] K, i.e. below the temperature of vitrification and in correspondence with the emergence of nearly hyperuniformity. In this temperature window, the sample is a glass and the topology of the HBN does not show any appreciable change; *P*(*n*)’s are peaked on *n* = 6 and the networks accommodate also *n* = 5, *n* = 7, and *n* = 8 rings. The absence of *n* = 4 is also particularly interesting. Squared rings are stiff configurations difficult to anneal at low temperature; they increase the local strain of the network and increase the fluctuations of bond angles decreasing the overall “quality” of the network (75). At these conditions, ${\sim}10\%$ of water molecules change one or more hydrogen bonded neighbor (Fig. 1b). Therefore, the kinetics of the HBN follows the same correlated kinetics described above and pictorially represented in Fig. 3.

**Fig. 4:**
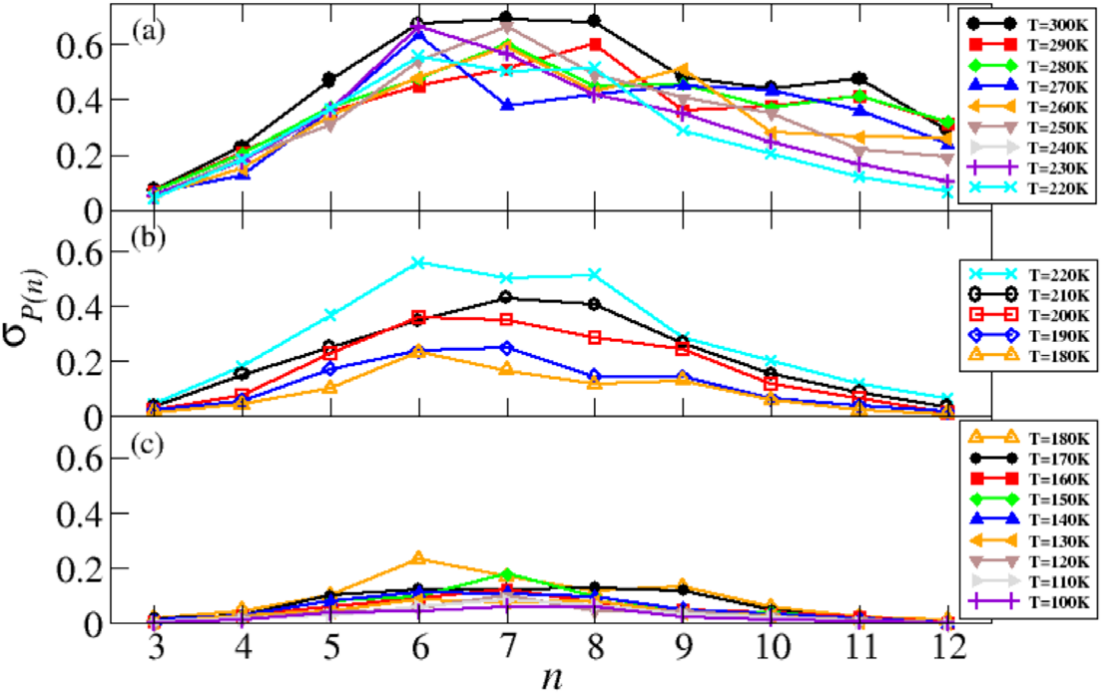
Fluctuations σ of the HBN reports σ in the temperature window *T* ∈ [300 − 220] K corresponding to the slow decrease in the index *H*. Panel (b) shows σ in the temperature window *T* ∈ [220 − 180] K corresponding to the enhanced reduction in the index *H*. Panel (c) reports σ in the temperature window *T* ∈ [180 − 100] K in correspondence of the emergence of nearly hyperuniformity.

The dynamic nature of the HBN that gives rise to density fluctuations can be quantified accessing the network’s fluctuations. In Fig. 4 we report σ, the value of the standard deviations of each ring in the *P*(*n*)’s reported in Fig. 2.

Panel (a) shows σ in the temperature window *T* ∈ [300 − 220] K, when the liquid is pervaded by large-scale density fluctuations. It is possible to appreciate the noisy, intense degree of fluctuations of the HBN. In particular, the degree of fluctuations at the level of long (*n* > 9) rings is as high as at the level of short (*n* ∈ [4, 5]) rings, indicating that the HBN as a whole undergoes pervasive rearrangements that cause density fluctuations.

Panel (b) reports σ in the temperature window *T* ∈ [220 − 180] K, when the value of *H* in the supercooled liquid decreases. The reduction of fluctuations in the HBN is here very noticeable, a temperature-dependent trend very clear. Fluctuations dampen strongly upon cooling and tend to become negligible on the few longer (*n* > 9) rings. This observation further demonstrates that the HBN suppresses large-scale density fluctuations in the supercooled liquid phase. Overall, the clear temperature-dependent reduction of σ explains the increased rate at which the index *H* decreases in this temperature window.

Panel (c) shows σ in the temperature window *T* ∈ [180 − 100] K, i.e. in correspondence of the glassy phase of water in which nearly hyperuniformity emerges. The fluctuations of the HBN are considerably damped, hence accounting for the reduced large-scale density fluctuations.

To delve deeper into the correlated nature of the HBN emerging at supercooled conditions, we inspect the “quality” of the HBN in terms of coordination defects. We focus on the simplest coordination defects, in which a water molecule has three HBs. Given a water molecule, two configuration give rise to this geometry, namely *A*_2_*D*_1_ (two acceptors “*A*” and one donor “*D*”), and *A*_1_*D*_2_ (one acceptor “*A*” and two donors “*D*”). Besides the perfectly four-folded coordinated case *A*_2_*D*_2_ characterizing a CRN, *A*_2_*D*_1_ and *A*_1_*D*_2_ defects represent the mostly occurring configurations in liquid water at ambient conditions (52,76). However, since the *A*_2_*D*_1_ defects occur with a frequency almost double than *A*_1_*D*_2_ defects in bulk water at ambient conditions (52,76), we focus on *A*_2_*D*_1_ configurations only.

**Fig. 5:**
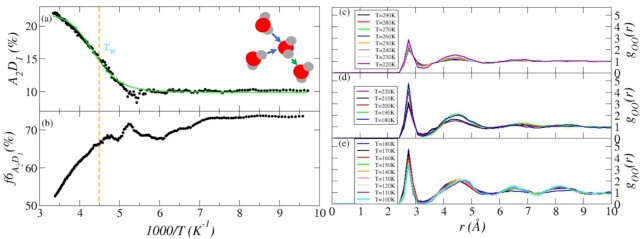
Panel (a): percentage-wise profile as a function of 1/*T* for *A*_2_*D*_1_ defects (black circles). The green line represents the fit with a four point logistic fit. The flex occurs roughly in correspondence with *T_W_*. Panel (b): percentage-wise profile as a function of 1/*T* for $f6_{A_2D_1}$, the percentage of *A*_2_*D*_1_defects involved in changes in local topology preserving hexagonal configurations. The snapshot represents the *A*_2_*D*_1_ geometry, with the central water molecules accepting two bonds emphasized by the blue arrows and donating one bond emphasized by the green arrow. Panel (c) reports the *g_OO_*(*r*)’s computed between oxygen atoms of *A*_2_*D*_1_ defects only in the temperature window *T* ∈ [300 − 220] K, panel (d) in the temperature window *T* ∈ [220 − 180] K, and and panel (e) in the temperature window *T* ∈ [180 − 100] K.

Upon cooling, the network of bonds gradually build up (19) and the percentage of coordination defects decreases. On the other hand, spatial and dynamical heterogeneities develop affecting transport properties (19, 22–24) and water molecules keep changing neighbors (Fig. 1b). We here posit that *A*_2_*D*_1_ defects represent the source of the decoupling between rotational and translational degrees of freedom leading to the breakdown of the Stokes–Einstein relation occurring at *T_W_*, and govern the kinetics of the HBN below *T_W_*. In Fig. 5(a), we report the percentage of *A*_2_*D*_1_ coordination defects upon cooling. As expected, the percentage of defects decreases with decreasing the temperature, but such reduction is not linear; rather, as for the profile of *C* (Fig. 1b), also the profile of *A*_2_*D*_1_ defects follows a logistic profile (green line) with a flex roughly located in correspondence of *T_W_*. Interestingly, the percentage of *A*_2_*D*_1_ defects drops in the temperature window 1,000/*T* ∈ [∼5.0 − 5.2] K^−1^ followed by a sudden increase. This increase is unexpected, considering that it occurs in the proximity of the temperature of dynamical arrest and, upon lowering the temperature, coordination defects should disappear. On the other hand, this increase explains and accounts for the sudden increase of the hyperuniformity index *H* reported in Fig. 1(a). Interestingly, although *A*_2_*D*_1_ defects decrease upon cooling, they acquire an increasingly pivotal role in the dynamics of the HBN. In Fig. 5(b), we report $f6_{A_2D_1}$, the percentage of *A*_2_*D*_1_ defects involved in the dynamics of hexagonal rings at every ps. We compute $f6_{A_2D_1}$ as follows: at a given time step *t* we list all hexagonal rings containing an *A*_2_*D*_1_ defect. After 1 ps, we count how many of these rings have preserved the hexagonal topology but exchanged the *A*_2_*D*_1_ defect with another water molecule. $f6_{A_2D_1}$ is the percentage of *A*_2_*D*_1_ defects involved in such mechanism. Remarkably, we can observe that $f6_{A_2D_1}$ increases from ${\sim}50\%$ at 1,000/*T* ∼ 3.5/K to ${\sim}65\%$ at *T* ∼ 1,000/*T* ∼ 4.5/K, to stabilize at ${\sim}75\%$ at lower temperatures. Therefore, considering that the overall dynamics of the HBN slows down (Fig. 1b) and that the percentage of *A*_2_*D*_1_ defects hosted in the network decreases upon cooling (Fig. 5a), the profile of $f6_{A_2D_1}$ allows us to conclude that *A*_2_*D*_1_ defects play a pivotal role in the kinetics of the HBN and, possibly, in the development of the dynamical heterogeneities below *T_W_*. Although the inspection of $f6_{A_2D_1}$ is not an exhaustive investigation of the many possible mechanisms underlying the overall kinetics of the HBN, it nonetheless highlights the central role of *A*_2_*D*_1_ defects in determining the complex behavior of water. Moreover, considering the central molecule in *A*_2_*D*_1_ defects has one dangling hydrogen, as pictorially reported in the snapshot in Fig. 5, we posit that rotations are relatively easier compared to translations; rotations could occur breaking only one of the HBs, compared to the breaking of three HB required to escape the cage of the neighbors. Therefore, we posit that *A*_2_*D*_1_ defects might be related to the supposed decoupling of translational and rotational degrees of freedoms occurring in correspondence with and below *T_W_*.

In order to delve deeper into the role of *A*_2_*D*_1_ defects, in Fig. 5(c)–(e) we report the two-body pair correlation function *g_OO_*(*r*) at different temperatures and computed between oxygen of *A*_2_*D*_1_ defects only. In panel (c), we report the *g_OO_*(*r*) in the temperature window *T* ∈ [300 − 220] K, i.e. when the HBN is strongly fluctuating. We can observe that the *g_OO_*(*r*)’s lack long-range spatial correlation, as one would expect for a liquid (as well as for a glass). On the other hand, the *g_OO_*(*r*)’s clearly deviates from that of a random distribution, that one would expect to observe in a uncorrelated system. At high temperatures, the *g_OO_*(*r*)’s are characterized by a low intensity first peak at ∼2.7 Å and a very mild second peak at ∼4.2 Å. Upon approaching *T_W_* = 220 K, we observe an enhancement of the second hydration peak and signatures of a third shell appearing at ∼6.7 Å. In panel (d), we report the *g_OO_*(*r*)’s in the temperature window *T* ∈ [220 − 180] K, i.e. when the HBN of the supercooled liquid allows an enhanced suppression of large-scale density fluctuations, and the overall topology acquires a steady-like configuration. We can observe the development of long-range (beyond the second shell of neighbors) correlations with hints of a fourth coordination shell emerging at ∼8.5 Å. Correspondingly, the interstitial space between first and second shells of neighbors become less populated. In panel (e) we report the *g_OO_*(*r*)’s in the temperature window *T* ∈ [180 − 100] K, i.e. in correspondence with the dynamical arrest and the emergence of nearly hyperuniformity. We can observe a further development of long-range spatial correlation and an enhanced structurization of the third and fourth hydration shells. Overall, our observations explain, end expand upon, the observations of clustered dynamical heterogeneities observed in supercooled water (22–24). Similar observations can be drawn inspecting the spatial correlation between *A*_1_*D*_2_ defects.

## Conclusions

We have performed large-scale out-of-equilibrium molecular dynamics simulations to model the cooling and vitrification of liquid water at ambient pressure. By tracking large-scale density fluctuations via the hyperuniformity index *H* and the HBN via the ring statistics, we have unveiled a hitherto hidden connection between the ability of suppressing large-scale density fluctuations and the topology of the HBN. At high temperatures the liquid sample is pervaded by large-scale density fluctuations, the HBN strongly fluctuates and the corresponding topology changes configurations. In correspondence with the Widom temperature (*T_W_* ∼ 220 K) the sample undergoes a crossover and below *T_W_* the *liquid* phase is endowed by a enhanced suppression of large-scale density fluctuations. Correspondingly, the fluctuations of the HBN are strongly reduced and the topology almost steady. The preserved topology, i.e. the roughly temperature-independent rings composition of the HBN and the concomitant development of long-range spatial correlations between coordination defects, signal the existence of cooperative bonds breaking / making mechanisms with long-range correlations that govern the kinetics of the HBN in the supercooled liquid. A detailed analysis of the dynamical and spatial behavior of *A*_2_*D*_1_ coordination defects indicates that these defects play a pivotal role in the complex kinetics of the HBN, and such role increases upon decreasing the temperature. We also posit that *A*_2_*D*_1_ defects may be the source of the decoupling between translational and rotational degrees of freedom occurring in correspondence with the fragile-to-strong transition. From an experimental perspective, deconvolving absorption spectra to identify the contribution of *A*_2_*D*_1_ configurations could help in verifying our hypothesis.

Nearly hyperuniformity emerges below the temperature of vitrification *T_v_*, as the fluctuations of the HBN are strongly suppressed and the topology of the HBN steady. Nonetheless, the network is dynamic and hosts a considerable amount of coordination defects. Inspecting the profile of structure factors obtained experimentally could help in validating our findings.

Rationalizing the concerted mechanisms underlying the HBN transformations in the supercooled liquid and in the glassy state is an alluring perspective (and a nontrivial task) that would deepen our understanding of the physics of supercooled liquids, and of water in particular. The concerted bond-switch mechanism initially proposed by Wooten et al. (77) to produce amorphous silicon and germanium (and related to the occurrence of Stone–Wales defects in 2D hyperuniform networks (78)) may be present, but they are only one of the several possible and more complex mechanisms involving longer-range correlations. Likewise, the discovery of nearly hyperuniformity in 3D networks hosting a considerable amount of coordination defects opens new avenues for the design of experimental set ups to make and test such kind of networks, as well as for better understanding the relationship between stiffness in 3D network forming materials and hyperuniformity.

## Data Availability

The data underlying this article are available upon request to the author.
